# Influence of N_2_ Partial Pressure on Structure and Mechanical Properties of TiAlN/Al_2_O_3_ Multilayers

**DOI:** 10.3390/ma6030795

**Published:** 2013-02-28

**Authors:** Jingyue Yan, Lei Dong, Chongkuan Gao, Ning Wang, Dejun Li

**Affiliations:** 1 College of Physics and Electronic Information Science, Tianjin Normal University, Tianjin 300387, China; E-Mails: yjydzu@163.com (J.Y.); dlei0008@126.com (L.D.); gao_chongkuan@163.com (C.C.); wnsunrain@126.com (N.W.); 2 Key Laboratory of Beam Technology and Material Modification of Ministry of Education, Beijing Normal University, Beijing 100875, China

**Keywords:** TiAlN/Al_2_O_3 _multilayers, magnetron sputtering, hardness, residual stress

## Abstract

TiAlN/Al_2_O_3 _multilayers with different Ar/N_2_ ratios were deposited on Sisubstrates in different N_2_ partial pressure by magnetron sputtering. The crystalline and multilayer structures of the multilayers were determined by a glancing angle X-ray diffractometer (XRD). A nanoindenter was used to evaluate the hardness, the elastic modulus and scratch scan of the multilayers. The chemical bonding was investigated by a X-ray Photoelectron Spectroscopy (XPS). The maximum hardness (36.3 GPa) and elastic modulus (466 GPa) of the multilayers was obtained when Ar/N_2_ ratio was 18:1. The TiAlN/Al_2_O_3_ multilayers were crystallized with orientation in the (111) and (311) crystallographic planes. The multilayers displayed stably plastic recovery in different Ar/N_2_ ratios. The scratch scan and post scan surface profiles of TiAlN/Al_2_O_3_ multilayers showed the highest critical fracture load (*L*_c_) of 53 mN for the multilayer of Ar/N_2_ = 18:1. It indicated that the multilayer had better practical adhesion strength and fracture resistance.

## 1. Introduction

The multi-element systems, in recent years, have received more attention in terms of further improving performance [1,2,3]. When applied properly, protective coatings on bearings, gears, cutting tools and other tribological components can extend component lifetimes. Though not universally true, it is generally desirable to have coatings with high hardness and low internal stress. Protective coatings with high hardness provide better wear resistance of coated steel tools against abrasion at high contact pressures [4]. Multilayer coatings should have low internal stress so that one can deposit thicker coatings without delamination. In this work, we focus our efforts on the optimization of process parameters to obtain these properties in multilayers. When individual layer thicknesses approach nanometer dimensions, the hardness of multilayers is generally enhanced over the rule-of-mixture values, typically by a factor of two. The enhancement is primarily due to the presence of interfaces [5]. In multilayers, interfaces are postulated to act as barriers against dislocation motion. There is ample evidence in the literature to support this postulate [6,7]. In addition, as the layer thickness and crystallite size approach nanometer dimensions, dislocation generation becomes energetically unfavorable. Both factors make multilayers stronger than expected from the rule of mixtures [8]. 

As is well-known, TiAlN coating is relatively excellent coating material due to its high hardness, wear, chemical inertness and superior oxidation resistance [9,10]. In addition to high hardness, low friction, and good adhesion, tools working under extreme conditions must possess high-temperature oxidation resistance. For example, the temperature at the tip of the tools can reach 1000 °C during high-speed machining. Nitride-based coatings are insufficient to survive such a high temperature resulting from oxidation. Most of the oxides, such as Al_2_O_3_ and SiO_2_, are much more stable at high temperature than nitrides. Unfortunately, oxides lack the strength that nitrides have to withstand the mechanical abuse of cutting. In practice, oxides have to be combined with nitride or carbide material, such as TiN or TiC, in the form of multilayered structure when being used in tool coatings. The nitride or carbide layer provides strength, while the oxide layer provides chemical resistance. Pronounced strength enhancement, optimal hardness/toughness ratios and excellent wear resistance can be obtained through a proper critical bilayer thickness design for nanoscale multilayers [11,12,13]. These coatings can be deposited by physical vapor deposition (PVD). PVD usually operates at lower temperatures, thus allowing a broader choice of substrates [14]. Films synthesized using PVD usually have compressive stresses [15]. 

## 2. Experimental Section 

TiAlN/Al_2_O_3 _multilayers with constant *Λ* (10 nm) and *t*_TiAlN_:* t*_Al2O3_ (10:1) were deposited on Si substrate at a fixed substrate bias of −140 V in different N_2_ partial pressure by magnetron sputtering. High purity Ti_3_Al (99.99%) target and Al_2_O_3_ (99.99%) target were respectively connected to RF source sputter guns, which were installed with the horizontal plane. First of all, Si (100) substrates were cleaned by an ultrasonic agitator with acetone and alcohol for at least 15 min and dried using compressed air after each cleaning cycle. Subsequently, all substrates were sputter-cleaned by −500 V bias for 10 min. The deposition of the multilayers started with the deposition of 80-nm-thick Ti buffer layer to improve coating adhesion. Then, the TiAlN/Al_2_O_3_ multilayers were fabricated in a flowing Ar and N_2_ gas mixture with different N_2_ partial pressure and the working pressure was kept at 0.4 Pa. The ratios of Ar/N_2_ were from 10:1 to 22:1. In the process of deposition, all the depositions were conducted using a power of 100 W for the Ti_3_Al target and Al_2_O_3 _target. The thickness of the monolayer was controlled by open time of baffle and calculated by multiplying the deposition rate by the sputtering time. All multilayers were deposited 30 periods. Total thicknesses of the multilayers were around 400~500 nm.

The element compositions, their depth profiles and chemical states of the coatings were investigated by a PHI5000 Versa Probe X-ray Photoelectron Spectroscopy (XPS, PHI5000, USA) using an Al Kα X-ray source (1486.6 eV). A D/MAX 2500 diffractometer was used for low-angle X-ray reflectivity (XRR) and wide angle X-ray diffraction (XRD) of layered structure and crystalline analysis, operating with Cu Kα radiation at 1.54056 Å. The nanohardness, the elastic modulus and loading-unloading curves of the multilayers were investigated by the continuous stiffness measurement technique using a Nano Indenter XP system. This system was also used to perform scratch test. Residual stress generated during the coating growth process was calculated according to the curvature measured using an XP-2 profiler.

## 3. Results and Discussion

### 3.1. Structural Characterizations 

#### 3.1.1. X-ray Diffraction Analysis

**Figure 1:**
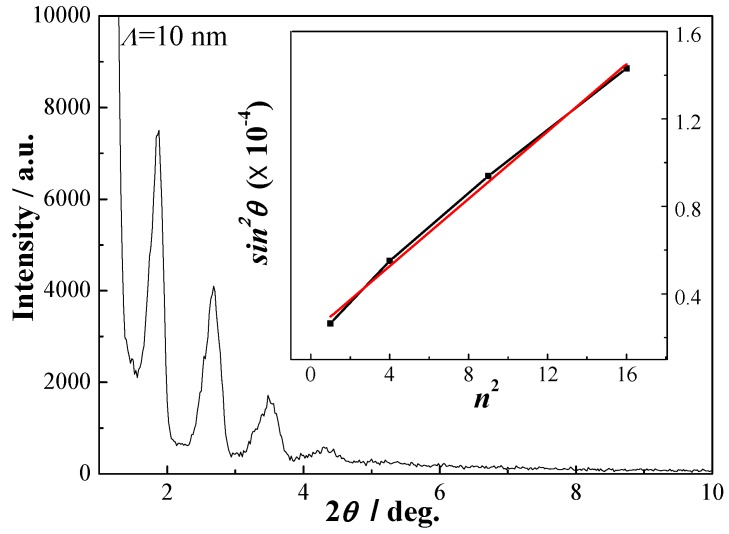
High resolution X-ray reflectivity (XRR) curve of TiAlN/Al_2_O_3_ multilayer (*Λ* = 10 nm).

Figure 1 shows the low-angle XRD patterns of the TiAlN/Al_2_O_3_ multilayers. Clear reflection peaks are observed in XRR pattern of TiAlN/Al_2_O_3_ multilayer, indicating that the multilayer has distinct chemical modulation structure and sharp interfaces. Sharp interfaces between the two layers throughout whole multilayer can cause an enhancement in hardness. The modulation period (*Λ* = 10 nm) is calculated according to the periodic position of the reflection peaks using the modified Bragg’s law [16]: ${\text{sin}}^{\text{2}}\theta_{n}=（\frac{n\lambda }{2\Lambda }{）}^{2}+2\delta$ , where n is the ordinal number of reflection peak, *λ* = 1.54056 Å is the X-ray wavelength, and δ is the correct value (1.749 × 10^−5^) which is related to the average reflective index. Using equation above, the *Λ* is calculated by the slope of linear least-squares fitted straight line of sin^2^*θ_n_ versus* *n*^2^ which is shown in the inserted figure.

**Figure 2:**
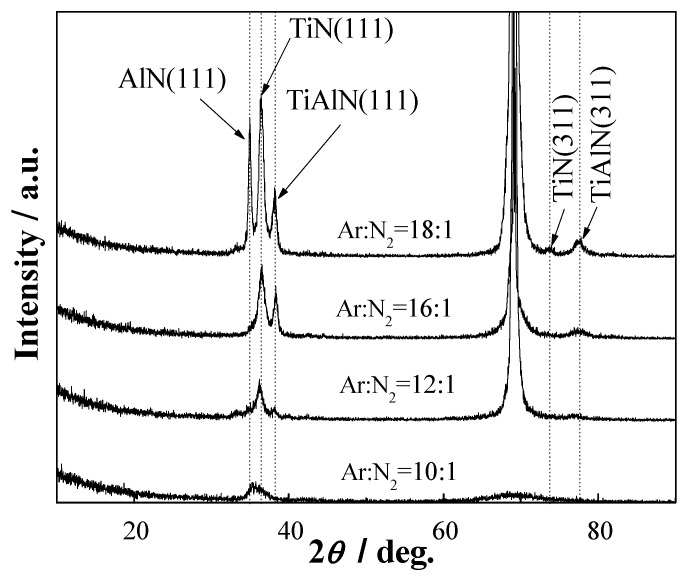
X-ray diffractometer (XRD) patterns of TiAlN/Al_2_O_3 _multilayers at different Ar/N_2_ ratios.

Figure 2 shows the XRD patterns of TiAlN/Al_2_O_3_ multilayers deposited at Si substrates with different Ar/N_2 _from 10:1 to 18:1. All the investigated multiayers were highly textured with a preferential orientation in the (111) direction, but the intensities of (311) orientation was very low. With increasing Ar/N_2_, TiAlN(111) and AlN(111) peaks began to appear, the intensity of three different phases (111) have reached maximum and become sharper, when Ar/N_2_ ratio is 18:1. It indicates that multilayer (Ar/N_2_ = 18:1) have larger grains and higher degree of crystallinity [17]. The radius of Al atom is 0.1440 nm, but the radius of Ti atom is 0.1461 nm. In TiAlN layer, the titanium atoms in TiN lattice were replaced by aluminum atoms. The different of atom radius led to distortion of lattice and changed internal stress of TiAlN/Al_2_O_3_ multilayers. The peak broadening behavior was usually originated from the diminution of grain size [18] and the existence of residual stress induced in the crystal lattice [19]. No reflection peaks assigned to Al_2_O_3_ phase is observed, meanwhile, there has also been the presence of Al_2_O_3_ in XPS. It indicates that Al_2_O_3 _layer is amorphous phases in all multilayers. This structure change is ascribed to grains growth induced by amorphous Al_2_O_3 _periodic insertion. The combined nanocrystallities and amorphous phases can produce a positive effect on mechanical properties.

#### 3.1.2. X-ray Photoelectron Spectroscopy Analysis

**Figure 3:**
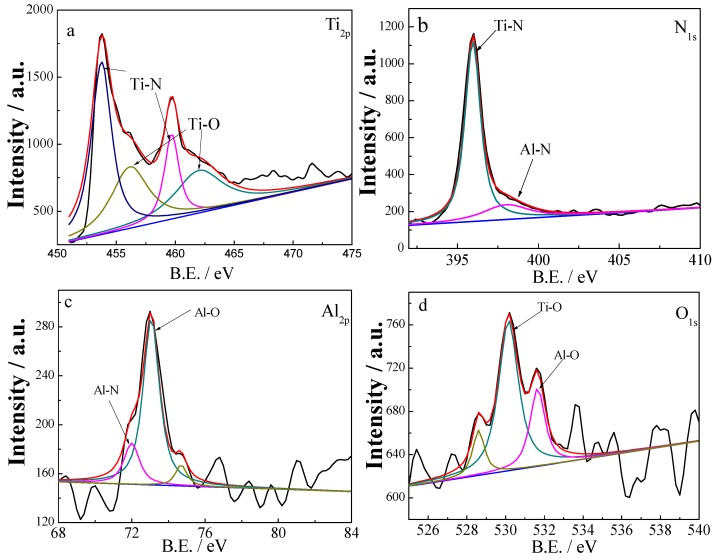
High resolution X-ray Photoelectron Spectroscopy (XPS) spectra of Ti_2p_, N_1s_, Al_2p_, O_1s_ electrons for the TiAlN/Al_2_O_3_ multilayer.

The high-resolution XPS core-level spectra of Ti_2p_, N_1s_, Al_2p_ and O_1s_ for TiAlN/Al_2_O_3_ multilayer are shown in Figure 3. The chemical bonding states are obtained by subtracting the background with the Shirley’s method and deconvoluting the spectra by a curve-fitting method using a non-linear least squares fitting to a mixed Gaussian–Lorentzian product function [20]. The bonding status of Ti is investigated and shown in Figure 3a, peaks with binding energy of 453.9 and 456.2 eV are assigned to Ti_2p3/2_ stoichiometric TiN and TiO*_x_*, respectively [21]. The binding energy of 459.7 eV and 462 eV are related to Ti_2p1/2_–N and Ti_2p1/2_–O. The N_1s_ peaks at 396 eV and 398 eV are related to Ti–N bonding and Al-N bonding in Figure 3b. The Al_2p_ spectrum, Figure 3c, has been tentatively identified with two components. This spectrum is composed of contributions of nitridic Al in AlN (BE = 72 eV) and oxidic Al in Al_2_O_3_ (BE = 72 eV). The rich aluminum oxide in multilayer undoubtedly provides better protection against the environment attack. Figure 3d reveals that O is present as TiO*_x_* (530 eV) and Al_2_O_3_ (531.5 eV). The existing of TiO*_x_* in deep layer might because Ti is preferred to combine with O more than N for its higher activity. This result is in agreement with that concluded from XRD tests. From XPS measurement and combined with our XRD results with no Al_2_O_3_ diffraction peaks, we concluded that Al_2_O_3_ was amorphous phase and the multilayers existed as nc-TiAlN/a-Al_2_O_3_. It can be conceived that the amorphous nature of Al_2_O_3_ enables it to imbed among TiAlN grains cohesively, which would contribute to the enhancement of the hardness of the TiAlN/Al_2_O_3_ multilayers.

### 3.2. Mechanical Properties 

#### 3.2.1. Hardness and Elastic Modulus Analysis

**Figure 4:**
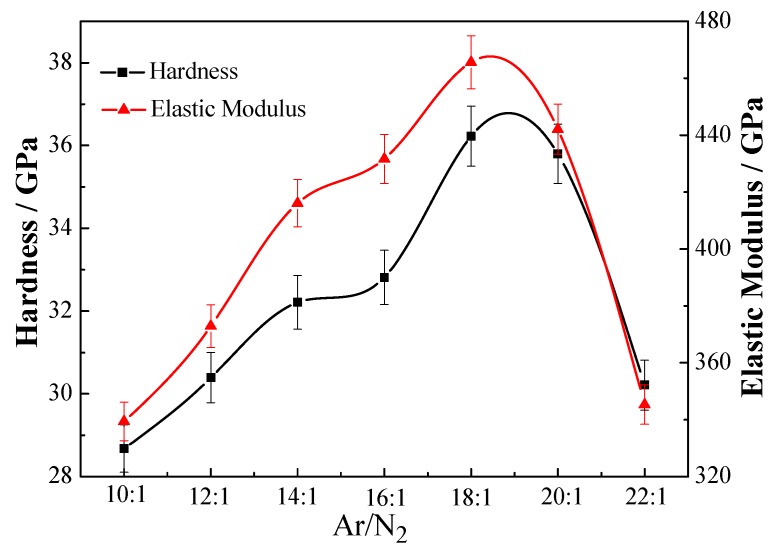
Hardness and elastic modulus of TiAlN/Al_2_O_3 _multilayers at different Ar/N_2_ ratios.

Figure 4 shows the hardness and elastic modulus of the TiAlN/Al_2_O_3_ multilayers with *Λ* ≈ 10 nm and different Ar/N_2_. With the increasing of Ar/N_2_ ratio, the hardness and elastic modulus of the multilayers all increase before decrease, the maximum hardness and elastic modulus of the multilayers are found to be 36 GPa and 466 GPa, respectively, when Ar/N_2_ ratio is 18:1. Excellent mechanical properties have come from TiAlN layer. Appropriate N_2_ is beneficial for TiAlN crystallinity, but an excess of N_2_ is to the disadvantage of Al_2_O_3_ layer formation. The increase in hardness and elastic modulus can be understood by studying the XRD patterns mentioned above. We believe that a strong mixture of TiAlN(111), AlN(111), TiN(111) and TiAlN(311) textures with larger grain sizes is responsible for hardness and elastic modulus increase. In addition, Hardness enhancement has been attributed to the periodic insertion of thinner amorphous Al_2_O_3_ into TiAlN related to suppressed dislocation generation and impeding dislocation motion. Interfaces act as barriers to the motion of dislocations and provide sites for dislocation pile-up [22].

#### 3.2.2. Residual Stress Analysis

**Figure 5:**
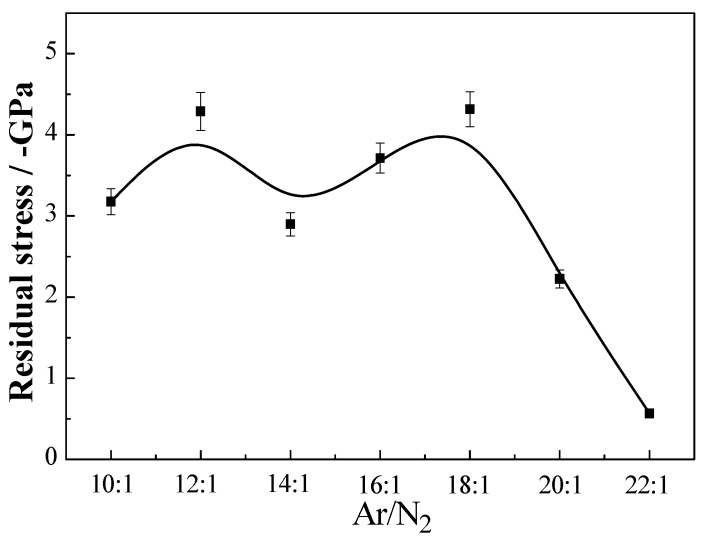
Residual stresses of TiAlN/Al_2_O_3 _multilayers at different Ar/N_2_ ratios.

The compressive stress of the multilayer coatings determined by XP-2 profiler is influenced by Ar/N_2_ ratios as is shown in Figure 5. It is known that residual stress is generated during the coating growth process. High residual stress (*σ*) is the main reason for coating delamination and plastic deformation. Therefore, the reduced residual stress in coatings is a key factor for these coatings to explore more applications. The residual stress calculated by the Stoney formula [23]: *σ* = −$\frac{E_{s}t_{s}^{2}}{6t_{c}(1-v_{s})R}$. Where *E*_s_, *t*_s_ and *v*_s_ are elastic modulus (131 GPa), thickness (0.0005 m) and Poisson’s ratio (0.28) of the substrate, separately; *t*_c_ is the coating thickness; and *R* is the radius of curvature of the multilayer coated on substrate. *R* can be determined by the film curvature using surface profilometry. The compressive stresses decrease with the increase of Ar/N_2_. When the Ar/N_2_ flow rate is 18:1, the compressive stress gets to the highest (−4.3 GPa) connected with highest hardness (36.3 GPa). Multilayers with Ar/N_2_ ratio more than 18:1 have lower compressive stress than others. It is due to periodically introduction of Al_2_O_3_ layers into TiAlN layers that helps to relax the stress.

#### 3.2.3. Loading-Unloading Curves Analysis

**Figure 6:**
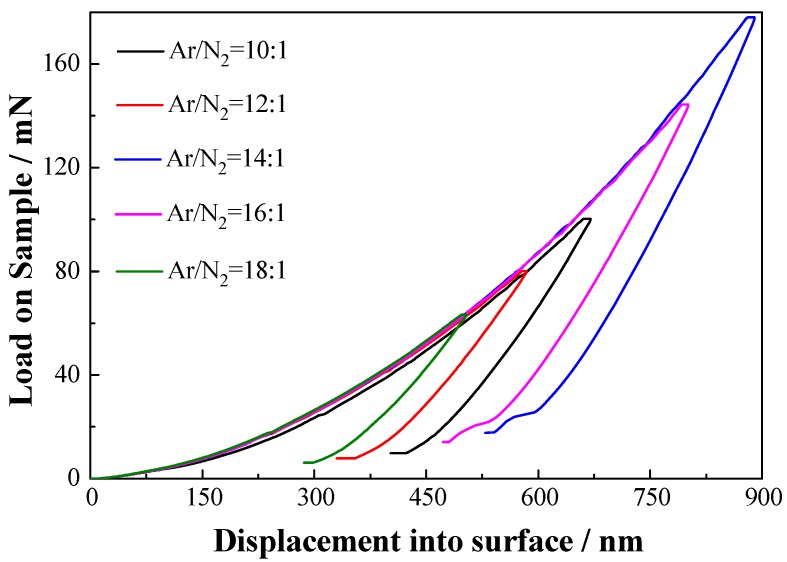
Typical load *vs*. nano-indenter displacement curve of TiAlN/Al_2_O_3_ multilayers with different Ar/N_2_ ratios

Typical observations of the loading-unloading curves on the different Ar/N_2_ are shown in Figure 6. All measured curves were obtained using a computer controlled stepwise increase of the load up to a chosen maximum loading (*L*_max_), followed by stepwise unloading. Usually, the elastic recovery (ER) is defined as ER = (*d*_max_ − *d*_res_)/*d*_max_, where *d*_max_ and *d*_res_ are the displacement at the maximum load and residual displacement after unloading, respectively [24]. Obviously, when pressed different depth of penetration into surface, loading-unloading curves of all TiAlN/Al_2_O_3_ multilayers are parallel. The value of (*d*_max_ − *d*_res_)/*d*_max_ is about 0.4. This indicates that plastic recovery (ER) has nothing to do with depth of penetration into surface. The stably plastic recovery (ER) is observed. With the change of Ar/N_2_, the plastic recovery displays invariable due to stably value of (*d*_max_ − *d*_res_)/*d*_max_.

#### 3.2.4. Scratch Test Analysis

**Figure 7:**
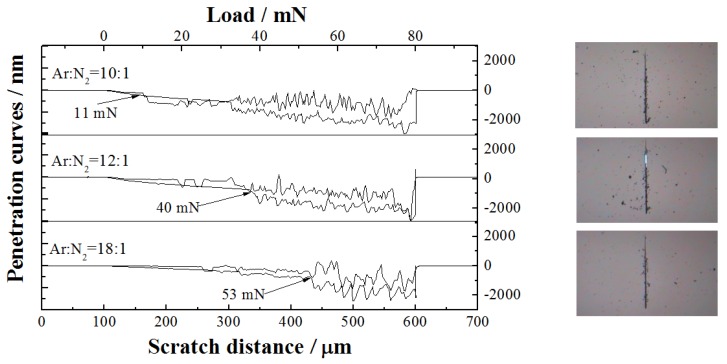
Surface profiles of the scratch-scan on TiAlN/Al_2_O_3_ multilayers with different Ar/N_2_ ratios.

Figure 7 shows the results of scratch test reflecting the fracture resistance of TiAlN/Al_2_O_3_ multilayers with different Ar/N_2_ ratios. The scratch scan profiles of all multilayers indicate an abrupt increase point in scratch depth. The normal load corresponding to an abrupt increase point in the scratch scan profile is the critical fracture load of coating (*L*_c_). The *L*_c_ can characterize the adhesion strength of the coating. The *L*_c_ of Ar/N_2_ = 10:1 and 12:1 are 11 and 40 mN, respectively. However, no abrupt change in scratch depth is observed until *L*_c_ = 53 mN for the multilayer of Ar/N_2_ = 18:1, which has better practical adhesion strength and fracture resistance. In terms of the above results, the multilayer’s fracture resistance may be interrelated with inherent internal stress, hardness, and plastic recovery of the coatings with multilayered structures.

## 4. Conclusions 

TiAlN/Al_2_O_3 _multilayers were deposited on Si substrate by magnetron sputtering. The effect of Ar/N_2_ ratios on the microstructure and properties of TiAlN/Al_2_O_3_ multilayers was investigated. With increasing Ar/N_2_, the intensity of (111) and (311) peaks becomes stronger. Meanwhile, the hardness and elastic modulus of the multilayers have all increased before decrease. When Ar/N_2_ ratio was 18:1, the maximum hardness and elastic modulus of the multilayers were found to be 36 and 466 GPa, respectively. The TiAlN/Al_2_O_3_ multilayer with Ar/N_2_= 18:1 has displayed better practical adhesion strength and fracture resistance. Therefore, the TiAlN/Al_2_O_3_ multilayers could be an effective and suitable candidate for hard coating system such as coated cutting tools. Such optimal processing parameters (Ar/N_2_= 18:1) would be quite favorable for industrial application in the future.
